# Synthesis and Biological Activities of Ethyl 2-(2-pyridylacetate) Derivatives Containing Thiourea, 1,2,4-triazole, Thiadiazole and Oxadiazole Moieties

**DOI:** 10.3390/molecules22030409

**Published:** 2017-03-06

**Authors:** Daniel Szulczyk, Piotr Tomaszewski, Michał Jóźwiak, Anna E. Kozioł, Tadeusz Lis, David Collu, Filippo Iuliano, Marta Struga

**Affiliations:** 1 Department of Biochemistry and Pharmacogenomics, Faculty of Pharmacy, Medical University of Warsaw, 02-097 Warsaw, Poland; michal.jozwiak@wum.edu.pl (M.J.); marta.struga@wum.edu.pl (M.S.); 2 Laboratory of Centre for Preclinical Research, Medical University of Warsaw, Banacha 1B, 02-097 Warsaw, Poland; 3 Department of Biochemistry, Second Faculty of Medicine, Medical University of Warsaw, 02-097 Warsaw, Poland; piotr.tomaszewski@wum.edu.pl; 4 Faculty of Chemistry, Maria Curie-Sklodowska University, 20-031 Lublin, Poland; anna.koziol@poczta.umcs.lublin.pl; 5 Faculty of Chemistry, University of Wroclaw, 50-383 Wroclaw, Poland; tadeusz.lis@chem.uni.wroc.pl; 6 Department of Life and Environmental Sciences, Section of Microbiology and Virology, University of Cagliari, 09042 Cittadella Universitaria Monserrato, Italy; david.collu@unica.it; 7 Department of Molecular Medicine, Institute of Virology, Slovak Academy of Sciences, Dubravska cesta 9, 845 05 Bratislava, Slovak; filippo.iuliano@unica.it

**Keywords:** 1,2,4-triazole, 1,3,4-thiadiazole, 1,3,4-oxadiazole, thiourea, X-ray crystal structure analysis, biological activity

## Abstract

Thirty six novel heterocyclic derivatives of ethyl 2-(2-pyridylacetate) were efficiently synthesized. The new compounds involve the linkage of a 2-pyridyl ring with thiosemicarbazide (compounds **1**–**7**), 1,2,4-triazole (compounds **1a**–**7a**), 1,3,4-thiadiazole (compounds **1b**–**7b**), and 1,3,4-oxadiazole (compounds **1f**–**7f**) moieties. The last group of compounds **1e**–**7e** involves the connection of a 2-pyridyl ring with 1,2,4-triazole and thiourea. ^1^H-NMR, ^13^C-NMR and MS methods were used to confirm the structures of the obtained derivatives. The molecular structures of **3**, **3b**, **7a** and **7f** were further confirmed by X-ray crystallography. All obtained compounds were tested in vitro against a number of microorganisms, including Gram-positive cocci, Gram-negative rods and *Candida albicans*. In addition, the obtained compounds were tested for cytotoxicity and antiviral activity against HIV-1.

## 1. Introduction

**Figure 1:**
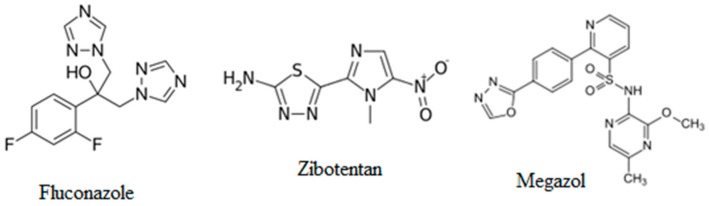
Active substances containing 1,2,4-triazole, 1,3,4-thiadiazole and 1,3,4-oxadiazole scaffold.

Heterocyclic compounds are well-known for their pharmaceutical importance, and the research for simple and efficient methods of synthesis of compounds incorporating heterocyclic rings has brought a new dimension to drug development [1]. In past decades heterocyclic scaffolds such as the 1,2,4-triazole, 1,3,4-oxadiazole and 1,3,4-thiadiazole rings and their derivatives were intensively investigated by researchers because of their biological activity. Among many heterocyclic compounds, these five-membered structures display unique spatial arrangements, which give possibilities to develop the synthetic pathways for microbiologically active compounds that can have potential therapeutic applications as new medicines. Currently, there are a lot of pharmaceutical products available on the market, which contain the previously mentioned structures such as fluconazole, efinaconazole, terconazole and fosfluconazole. These are used as antifungal agents and their structures are based on the 1,2,4-triazole core. The 1,3,4-thiadiazole scaffold can be found in other active substances like acetazolamide, methazolamide and megazol. Finally, the 1,3,4-oxadiazole arrangement is present in raltegravir and zibotentan. Exemplary structures of some of the previously listed medicines containing mentioned active scaffolds are presented in Figure 1 below.

Currently researchers continue to design and sythesize new 1,2,4-triazole, 1,3,4-oxadiazole and 1,3,4-thiadiazole derivatives because of their high antimicrobial activity against a wide range of Gram-negative and Gram-positive bacteria and yeasts. Compounds that contain one of mentioned heterocyclic motifs are often inhibitory or lethal against different types of microorganisms. Derivatives containing the 1,2,4-triazole core are still intensively studied as they possess potent antifungal properties [2]. Recently, 1,2,4-triazole-based compounds were reported as potential anticonvulsant [3,4,5,6,7] and anti-HIV agents [8,9]. Among the thiadiazole derivatives, those synthesized using the 1,3,4-thiadiazole motif were found to have number of biological activities such as anticonvulsant [10,11], antihypertensive [12,13], local anesthetic [14], anticancer [15,16], anti-HIV [17], anti-inflammatory [18,19], and hypoglycemic activities [20]. 1,3,4-Oxadiazoles are intensively evaluated for their activity against HIV-1 and HIV-2 in MT-4 cells [21,22,23,24].

All three scaffolds are fundamental in designing novel antimicrobial agents. Compounds built on these heterocyclic cores represent the non-steroidal antimicrobial drugs (NSAMDs). A study of the literature confirms that 1,3,4-oxadiazoles, 1,3,4-thiadiazoles, 1,2,4-triazoles and their amino derivatives are recognized as promising candidates for medicines used against bacteria and yeast [25,26,27,28,29]. These compounds often show higher activity than standard antibiotics commonly used as reference materials. Available data presume that the introduction of halogen atoms into the pharmacophore structure can be favourable for preventing the resistant microorganisms from spreading and emerging. These kinds of structural modifications help to increase the lipid-solubility of the studied scaffolds [30,31]. Ureas, thioureas, hydrazones, semicarbazides or thiosemicarbazides are found to posses similar properties and are useful as starting material in the synthesis of the three title heterocyclic arrangements and their structural modification. Prompted by this fact and encouraged by the results of recent studies [32,33,34] we decided to design synthetic paths to produce a battery of 1,3,4-oxadiazole, 1,3,4-thiadiazole, and 1,2,4-triazole derivatives.

## 2. Results and Discussion

### 2.1. Chemistry

**Scheme 1:**
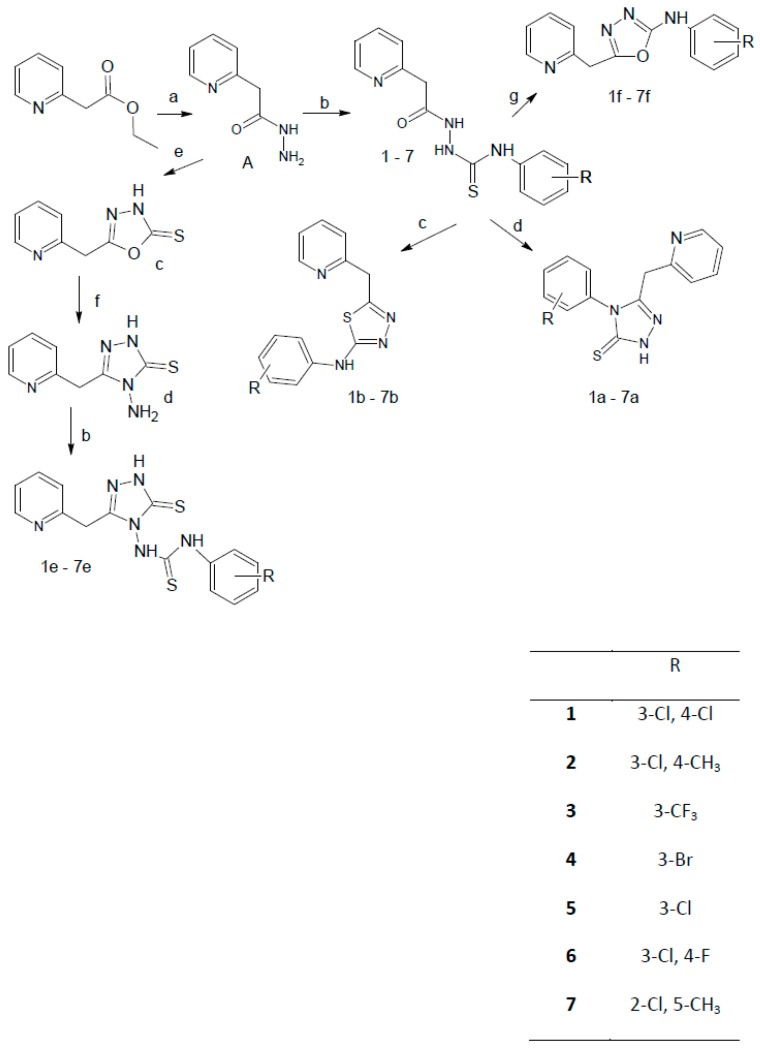
Synthesis of ethyl 2-(2-pyridylacetate) derivatives. *Reagents and conditions*: (**a**) NH_2_NH_2_ 80%, EtOH, rt. 2 h (**b**) appropriate isothiocyanate, acetonitrile, reflux, 6 h (**c**) H_2_SO_4(conc.)_ (**d**) 2% NaOH, reflux, 1 h (**e**) KOH, EtOH, CS_2_ (**f**) NH_2_NH_2_ 80%, reflux (**g**) Et_3_N, HgCl_2_, DMF.

Our aim was to obtain a small library of ethyl 2-(2-pyridylacetate) derivatives. The planned synthetic route is depicted in Scheme 1.

In first step the ester ethyl 2-(2-pyridylacetate) was transformed into 2-(pyridin-2-yl)acetohydrazide (A) [35,36]. This was a starting material to obtain the 2-pyridylacetate thiosemicarbazide and oxazole derivatives. Derivatives of *N*-(phenylsubstituted)-2-(pyridin-2-yl acetyl) hydrazinecarbothioamide (thiosemicarbazide derivatives) were prepared by reaction of hydrazide (A) with an appropriate aryl isothiocyanate. Briefly, 2-(pyridin-2-yl)-acetohydrazide (A) and different isothiocyanates were allowed to react in boiling acetonitrile for 6 h. *N*-(Phenylsubstituted)-2-(pyridin-2-yl-acetyl)hydrazinecarbothioamide derivatives **1**–**7** were the starting materials to obtain the corresponding 1,2,4-triazole, 1,3,4-thiadiazole and 1,3,4-oxadiazole derivatives **1a**–**7a**, **1b**–**7b** and **1f**–**7f**, respectively.

The methods for obtaining the 1,2,4-triazole and 1,3,4-thiadiazole derivatives **1a**–**7a** and **1b**–**7b** was similar. For both groups of compounds this required cyclisation, however for 1,2,4-triazole process was accomplished under basic conditions (2% NaOH) while for 1,3,4-thiadiazoles acidic conditions (conc. H_2_SO_4_) were used, respectively [37,38].

2-(Pyridin-2-yl)acetohydrazide (A) was also used to obtain 5-(pyridin-2-ylmethyl)-1,3,4-oxadiazole-2(3*H*)-thione (c). This compound was known [39], but in the present work a different synthesis method was applied. Hydrazide A was added to the solution of ethanol and KOH and next carbon disulfide was added to the mixture. The reaction mixture was refluxed on a steam bath for 5 h, then the solid formed was filtered off and recrystallized from butanol. 1,3,4-Oxadiazole-2-thione (c) was transformed into 4-amino-5-(pyridin-2-ylmethyl)-2,4-dihydro-3*H*-1,2,4-triazole-3-thione (d) by reaction with hydrazine hydrate. The free amino group offered a possibility for further reaction with isothiocyanates to obtain the thiourea derivatives **1e**–**7e**. This reaction was performed in acetonitrile similar to previously reported thiourea derivative syntheses [32,34]. 

**Figure 2:**
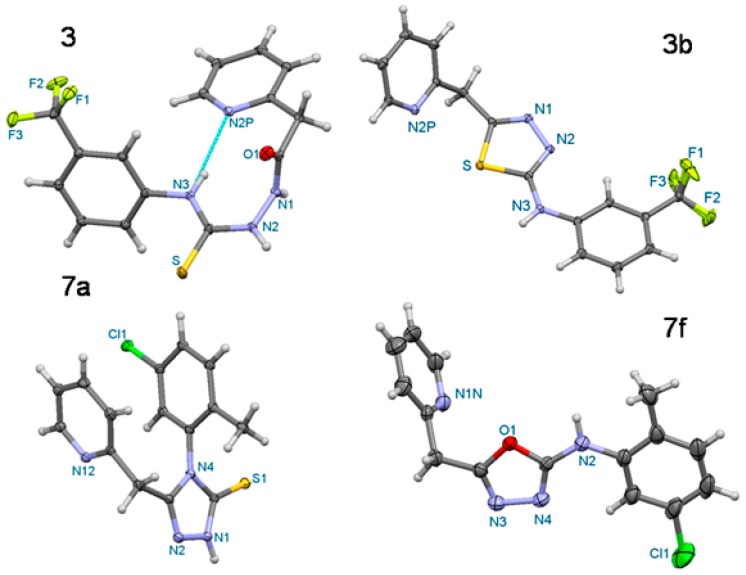
Perspective view of the molecular structures of the derivatives 2-(pyridin-2-yl-acetyl)-*N*-[3-(trifluoromethyl)phenyl]hydrazinecarbothioamide (**3**), 5-(pyridin-2-yl-methyl)-*N*-[3-(trifluoromethyl)phenyl]-1,3,4-thiadiazol-2-amine (**3b**), 4-(5-chloro-2-methylphenyl)-5-(pyridin-2-yl-methyl)-2,4-dihydro-3*H*-1,2,4-triazole-3-thione (**7a**) and *N*-(2-chloro-5-methylphenyl)-5-(pyridin-2-ylmethyl)-1,3,4-oxadiazol-2-amine (**7f**).

Oxadiazoles **1f**–**7f** were prepared according to a new procedure developed by our team. This synthesis relies on a desulfurization/cyclization reaction of thiosemicarbazides to oxadiazoles using mercuric chloride. Development of this synthetic procedure was inspired by a method already used for preparation of a library of amino acid derived 2-arylamino-[1,3,4]-oxadiazoles [40]. The mechanism of this type of reaction is already established, therefore our goal was to find suitable reaction conditions. It was found that reflux is not needed, furthermore catalytic amounts of triethylamine (1–3 drops) are optional. Applications of this synthetic route will be presented in future publications. The molecular structures of one of the parent derivatives, compound **3**, and three representatives of the cyclization products (**3b**, **7a** and **7f**) are illustrated in Figure 2. The non-covalent intermolecular contacts in the crystals are shown in the Supplementary Materials. The crystal data and experimental parameters are given in Table 1, below.

### 2.2. Biological Studies

All obtained compounds were tested in vitro against a number of bacteria, including Gram-positive cocci, Gram-negative rods and fungi. Microorganisms used in this study have common applications in the antimicrobial tests for many substances like antibiotics, antiseptic drugs and in the search for new antimicrobial agents [32,33,34,41]. The procedure started with a preliminary screening by the disc diffusion method, in order to select derivatives with antimicrobial properties. All obtained compounds are inactive in microbiological screening.

With the aim to evaluate more widely the biological properties of synthesized compounds (**1**–**7**, **1a**–**7a** and **1b**–**7b**) on the basis of reported anti-HIV activities of the similar derivatives, compounds were tested in cell-based assay against the human immunodeficiency virus type-1 (HIV-1), using efavirenz as reference inhibitor. The cytotoxicity against the MT-4 cells was evaluated in parallel with the antiviral activity (Table 2). None of the tested compounds showed selective anti-HIV-1 activity. Almost all tested compounds are non-cytotoxic for exponentially growing MT-4 cells (CC_50_ >100 µM).

## 3. Materials and Methods

### 3.1. Apparatus, Materials and Analysis

The NMR spectra were recorded on an AVANCE DMX 400 spectrometer (Bruker, Billerica MA, USA) operating at 300 MHz (^1^H-NMR) and 75 MHz (^13^C-NMR). The chemical shift values are expressed in ppm relative to TMS used as an internal standard. Mass spectral ESI measurements were carried out on ZQ Micromass instrument (Waters, Milford, MA, USA) equipped with a quadrupole mass analyzer. The spectra were run in the positive ion mode at a declustering potential of 40–60 V. The samples were previously separated on a UPLC column (C18) using an UPLC ACQUITY™ system by Waters connected with a DPA detector. Flash chromatography was performed on silica gel 60 (200–400 mesh, Merck, Kenilworth, NJ, USA) using chloroform/methanol (19:1 vol) mixture as eluent. Analytical TLC was carried out on silica gel F_254_ (Merck) plates (0.25 mm thickness). The diffraction data for **3** and **3b** were collected on an Xcalibur instrument (Thermo Fisher Scientific, Waltham, MA, USA), while that for **7a** and **7f** was collected on a SuperNova diffractometer (Agilent Technologies, Santa Clara, CA, USA). The CRYSALIS program system [42] was used for data collection, cell refinement and data reduction. The data were corrected for Lorentz and polarization effects. A multi-scan absorption correction was applied. The structure was solved using direct methods using the SHELXS-97, and refined by the full-matrix least-squares on *F*^2^ with the SHELXL-97 program [43]. All non-H atoms were refined with anisotropic displacement parameters. The H-atoms attached to carbon were positioned geometrically and refined using the riding model with *U*_iso_(H) = 1.2*U*_eq_(C). The nitrogen bonded H-atoms were found in the difference-Fourier map and refined with isotropic displacement parameters. The crystal data and experimental parameters used in the X-ray structure analysis are given in Table 1.

### 3.2. General Procedure of the Synthesis of N-(Phenylsubstituted)-2-(pyridin-2-ylacetyl)hydrazinecarbo-thioamide Derivatives 

2-(Pyridin-2-yl)acetohydrazide (A 0.01 mol, 1.51 g) and 0.01 mol of an appropriate isothiocyanate were refluxed in acetonitrile for 6 h. The product was filtered and washed carefully with diethyl ether in order to remove unreacted isothiocyanate, and then with water in order to remove hydrazide residues. The product was dried and then crystallized from an ethanol solution.

*N-(3,4-Dichlorophenyl)-2-(pyridin-2-ylacetyl)hydrazinecarbothioamide* (**1**). Yield 85%. m.p. 173–174 °C. ^1^H-NMR (DMSO-*d*_6_) δ (ppm): 3.72 (s, 2H, CH_2_); 7.24 (t, 1H, CH_arom._, *J* = 6.20 Hz); 7.37 (d, 1H, CH_arom._, *J* = 7.60 Hz); 7.44–7.46 (dd, 1H, CH_arom._, *J*_1_ = 3.46 Hz, *J*_2_ = 1.50 Hz); 7.59 (d, 1H, CH_arom._, *J* = 8.80 Hz); 7.74 (t, 1H, CH_arom._, *J* = 7,40 Hz), 7.80 (s, 1H, CH_arom._); 8.33 (s, 1H, CH_arom._); 9.95 (s, 1H, NH); 9.98 (s, 1H, NH); 10.32 (s, 1H, NH). ^13^C-NMR (DMSO-*d*_6_) δ (ppm): 42.73, 122.17, 124.18, 125.46, 127.0, 130.08 (2C), 130.26, 136.9, 139.31, 148.8, 155.65, 168.82, 180.72. ESI MS: *m*/*z* = 377.00 [M + Na] (100%).

*N-(3-Chloro-4-methylphenyl)-2-(pyridin-2-ylacetyl)hydrazinecarbothioamide* (**2**). Yield 83%. m.p. 183–184 °C. ^1^H-NMR (DMSO-*d*_6_) δ (ppm): 2.36 (s, 3H, CH_3_), 3.90 (s, 2H, CH_2_); 7.21 (t, 1H, CH_arom._, *J* = 6.20 Hz); 7.30 (d, 1H, CH_arom._, *J* = 7.60 Hz); 7.20 (m, 2H, CH_arom._); 7.52 (s, 1H, CH_arom._); 7.72 (t, 1H, CH_arom._, *J* = 7,40 Hz); 8.40 (s, 1H, CH_arom._); 9.19 (s, 1H, NH); 9.21 (s, 1H, NH); 10.30 (s, 1H, NH). ^13^C-NMR (DMSO-*d*_6_) δ (ppm): 19.07, 42.69, 122.16, 124.19, 125.71, 130.66, 132.27, 136.94, 138.22 (2C), 148.74 (2C), 155.76, 168.73, 180.72. ESI MS: *m*/*z* = 357.00 [M + Na] (100%).

*2-(Pyridin-2-ylacetyl)-N-[3-(trifluoromethyl)phenyl]hydrazinecarbothioamide* (**3**). Yield 72%. m.p. 177–178 °C. ^1^H-NMR (DMSO-*d*_6_) δ (ppm): 3.94 (s, 2H, CH_2_); 7.32 (d, 1H, CH_arom._, *J* = 8.00 Hz); 7.50 (m, 2H, CH_arom._); 7.70 (s, 2H, CH_arom._); 8.39 (d, 1H, CH_arom._, *J* = 4.00 Hz); 9.49 (s, 1H, NH); 9.52 (s, 1H, NH); 10.30 (s, 1H, NH). ^13^C-NMR (DMSO-*d*_6_) δ (ppm): 42.75, 118.63 (q) 121.67 (q, *J* = 3.70 Hz), 122.14, 124.2, 125.85 (q, *J* = 272.80 Hz), 128.63 (q, *J* = 33.40 Hz), 129.42, 168.86, 180.84. ESI MS: *m*/*z* = 377.00 [M + Na] (100%).

*N-(3-Bromophenyl)-2-(pyridin-2-ylacetyl)hydrazinecarbothioamide* (**4**). Yield 80%. m.p. 176–177 °C. ^1^H-NMR (DMSO-*d*_6_) δ (ppm): 3.76 (s, 2H, CH_2_); 7.35–7.49 (m, 5H, CH_arom._); 7.75–7.80 (m, 2H, CH_arom._); 8.35 (d, 1H, CH_arom._, *J* = 4.00 Hz); 9.49 (s, 1H, NH); 9.96 (s, 1H, NH); 10.33 (s, 1H, NH). ^13^C-NMR (DMSO-*d*_6_) δ (ppm): 42.72, 120.59, 121.82, 122.16, 124.19, 127.87, 130.16 (2C), 136.92, 140.76, 148.75, 155.72, 148.76, 168.79, 180.68. ESI MS: *m*/*z* = 366.30 [M + H] (100%).

*N-(3-Chlorophenyl)-2-(pyridin-2-ylacetyl)hydrazinecarbothioamide* (**5**). Yield 78%. m.p. 171–172 °C. ^1^H-NMR (DMSO-*d*_6_) δ (ppm): 3.76 (s, 2H, CH_2_); 7.25–7.44 (m, 5H, CH_arom._); 7.64 (br. s, 1H, CH_arom._); 7.78 (tt, 1H, CH_arom._, *J* = 4.56 Hz); 8.35 (d, 1H, CH_arom._, *J* = 4.00 Hz); 9.90 (s, 1H, NH); 9.97 (s, 1H, NH); 10.34 (s, 1H, NH). ^13^C-NMR (DMSO-*d*_6_) δ (ppm): 42.71, 122.15, 124.18, 124.98, 129.87, 132.25, 136.9, 140.63 (2C), 148.76 (2C), 155.72, 168.77, 180.73. ESI MS: *m*/*z* = 319.30 [M − H] (100%).

*N-(3-Chloro-4-fluorophenyl)-2-(pyridin-2-ylacetyl)hydrazinecarbothioamide* (**6**). Yield 72%. m.p. 188–189 °C. ^1^H-NMR (DMSO-*d*_6_) δ (ppm): 3.76 (s, 2H, CH_2_); 7.25–7.29 (dd, 1H, CH_arom._, *J*_1_ = 5.10 Hz, *J*_2_ = 1.50 Hz); 7.41 (m, 3H, CH_arom._); 7.71 (d, 1H, CH_arom._, *J* = 6.80 Hz); 7.78 (tt, 1H, CH_arom._, *J* = 4.56 Hz); 8.34 (d, 1H, CH_arom._, *J* = 4.00 Hz); 9.91 (s, 1H, NH); 9.93 (s, 1H, NH); 10.33 (s, 1H, NH). ^13^C-NMR (DMSO-*d*_6_) δ (ppm): 42.7, 116.51, 118.66, 122.15, 124.18, 126.17, 127.58, 136.36, 148.77 (2C), 153.05, 155.69, 168.78, 180.99. ESI MS: *m*/*z* = 337.30 [M − H] (100%).

*N-(5-Chloro-2-methylphenyl)-2-(pyridin-2-ylacetyl)hydrazinecarbothioamide* (**7**). Yield 78%. m.p. 192–193 °C. ^1^H-NMR (DMSO-*d*_6_) δ (ppm): 2.24 (s, 3H, CH_3_), 3.90 (s, 2H, CH_2_); 7.18–7.22 (dd, 1H, CH_arom._, *J*_1_ = 4.53 Hz, *J*_2_ = 1.80 Hz); 7.30 (d, 1H, CH_arom._, *J* = 10.80 Hz); 7.2 (t, 1H, CH_arom._, *J* = 8.40 Hz); 7.62 (d, 1H, CH_arom._, *J* = 10.00 Hz); 7.52 (tt, 1H, CH_arom._, *J* = 4.48 Hz); 8.2 (d, 1H, CH_arom._, *J* = 2.80 Hz); 8.40 (d, 1H, CH_arom._, *J* = 4.00 Hz); 9.18 (s, 1H, NH); 9.21 (s, 1H, NH); 10.30 (s, 1H, NH). ^13^C-NMR (DMSO-*d*_6_) δ (ppm): 17.05, 43.07, 122.2, 124.19, 126.67, 128.57, 129.5, 131.68, 134.82, 136.98, 139.3, 148.83, 156.16, 168.7, 181.47. ESI MS: *m*/*z* = 357.00 [M + Na] (100%).

### 3.3. General Procedure for the Synthesis of 5-(Pyridin-2-ylmethyl)-2,4-dihydro-3H-1,2,4-triazole-3-thione Derivatives

*N*-(Substituted-phenyl)-2-(pyridin-2-ylacetyl)hydrazinecarbothioamide (0.01 mol) was refluxed with 2% NaOH solution (40–50 mL) for 4 h. After cooling the solution was neutralized with dilute hydrochloric acid. The precipitated compound was filtered and then crystallized from ethanol.

*4-(3,4-Dichlorophenyl)-5-(pyridin-2-ylmethyl)-2,4-dihydro-3H-1,2,4-triazole-3-thione* (**1a**). Yield 79%. m.p. 192–193 °C. ^1^H-NMR (DMSO-*d*_6_) δ (ppm): 4.13 (s, 2H, CH_2_); 7.08 (d, 1H, CH_arom._, *J* = 10.40 Hz); 7.19 (dd, 1H, CH_arom._, *J*_1_ = 5.20 Hz, *J*_2_ = 2.40 Hz); 7.28 (d, 1H, CH_arom._, *J* = 4.93 Hz); 7.57 (d, 1H, CH_arom._, *J* = 3.20 Hz); 7.61 (tt, 1H, CH_arom._, *J* = 4.08 Hz), 7.68 (d, 1H, CH_arom._, *J* = 11.20 Hz); 8.37 - 8.39 (dd, 1H, CH_arom._, *J*_1_ = 4.80 Hz, *J*_2_ = 0.60 Hz); 13.88 (s, 1H, NH). ^13^C-NMR (DMSO-*d*_6_) δ (ppm): 33.99, 122.15, 123.41, 128.88, 130.51, 130.93, 131.34, 132.28, 133.46, 136.64, 148.99, 150.25, 154.59, 167.82. ESI MS: *m*/*z* = 361.00 [M + Na] (100%).

*4-(3-Chloro-4-methylphenyl)-5-(pyridin-2-ylmethyl)-2,4-dihydro-3H-1,2,4-triazole-3-thione* (**2a**). Yield 75%. m.p. 188–189 °C. ^1^H-NMR (DMSO-*d*_6_) δ (ppm): 2.32 (s, 3H, CH_3_); 4.07 (s, 2H, CH_2_); 7.07 (d, 1H, CH_arom._, *J* = 10.40 Hz); 7.11–7.12 (dd, 1H, CH_arom._, *J*_1_ = 8.10 Hz, *J*_2_ = 1.80 Hz); 7.17–7.21 (dd, 1H, CH_arom._, *J*_1_ = 7.20 Hz, *J*_2_ = 2.10 Hz); 7.28 (d, 1H, CH_arom._, *J* = 2.40 Hz); 7.38 (d, 1H, CH_arom._, *J* = 10.80 Hz); 7.61 (tt, 1H, CH_arom._, *J* = 4.56 Hz); 8.39 (d, 1H, CH_arom._, *J* = 4.80 Hz); 13.81 (s, 1H, NH). ^13^C-NMR (DMSO-*d*_6_) δ (ppm): 19.31, 34.01, 122.12, 123.33, 127.03, 128.58, 131.41, 132.43, 133.15, 136.6, 136.96, 148.97, 150.36, 154.72, 167.90. ESI MS: *m*/*z* = 339.00 [M + Na] (100%).

*5-(Pyridin-2-ylmethyl)-4-[3-(trifluoromethyl)phenyl]-2,4-dihydro-3H-1,2,4-triazole-3-thione* (**3a**). Yield 70%. m.p. 178–179 °C. ^1^H-NMR (DMSO-*d*_6_) δ (ppm): 4.13 (s, 2H, CH_2_); 6.99 (d, 1H, CH_arom._, *J* = 10.40 Hz); 7.13–7.17 (dd, 1H, CH_arom._, *J*_1_ = 6.60 Hz, *J*_2_ = 1.50 Hz); 7.52–7.64 (m, 4H, CH_arom._); 7.77 (d, 1H, CH_arom._, *J* = 9.60 Hz); 8.35 (d, 1H, CH_arom._, *J* = 4.80 Hz); 13.90 (s, 1H, NH). ^13^C-NMR (DMSO-*d*_6_) δ (ppm): 34.08, 118.01 (q), 121.62 (q, *J* = 4.20 Hz), 122.07, 123.29, 125.6 (q, *J* = 272.60 Hz), 126. 03 (q, 30.70 Hz) 129.51 (q, 30.80 Hz), 134.41, 136.6, 154.56, 167.91. ESI MS: *m*/*z* = 359.00 [M + Na] (100%).

*4-(3-Bromophenyl)-5-(pyridin-2-ylmethyl)-2,4-dihydro-3H-1,2,4-triazole-3-thione* (**4a**). Yield 79%. m.p. 222–223 °C. ^1^H-NMR (DMSO-*d*_6_) δ (ppm): 4.09 (s, 2H, CH_2_); 7.02 (d, 1H, CH_arom._, *J* = 10.40 Hz); 7.17–7.21 (dd, 1H, CH_arom._, *J*_1_ = 6.60 Hz, *J*_2_ = 1.80 Hz); 7.27 (m, 1H, CH_arom._); 7.36 (t, 1H, CH_arom._, *J* = 10.80 Hz); 7.42 (t, 1H, CH_arom._, *J* = 2.40 Hz); 7.57–7.62 (m, 2H, CH_arom._); 8.38 (d, 1H, CH_arom._, *J* = 4.80 Hz); 13.83 (s, 1H, NH). ^13^C-NMR (DMSO-*d*_6_) δ (ppm): 34.04, 121.24, 122.11, 123.34, 127.55, 130.87, 131.15, 132.24, 134.95, 136.62, 148.96, 150.27, 154.59, 167.82. ESI MS: *m*/*z* = 347.20 [M] (100%).

*4-(3-Chlorophenyl)-5-(pyridin-2-ylmethyl)-2,4-dihydro-3H-1,2,4-triazole-3-thione* (**5a**). Yield 73%. m.p. 223–224 °C. ^1^H-NMR (DMSO-*d*_6_) δ (ppm): 4.10 (s, 2H, CH_2_); 7.03 (d, 1H, CH_arom._, *J* = 10.40 Hz); 7.16–7.23 (m, 2H, CH_arom._); 7.31 (t, 1H, CH_arom._, *J* = 2.40 Hz); 7.39–7.50 (m, 2H, CH_arom._); 7.60 (tt, 1H, CH_arom._, *J* = 4.56 Hz); 8.38 (d, 1H, CH_arom._, *J* = 5.20 Hz); 13.84 (s, 1H, NH). ^13^C-NMR (DMSO-*d*_6_) δ (ppm): 34.04, 119.93, 122.14, 123.41, 127.19, 130.59, 133.1, 134.89, 136.61, 149.01, 150.3, 154.6, 167.98. ESI MS: *m*/*z* = 334.90 [M + Na] (100%).

*4-(3-Chloro-4-fluorophenyl)-5-(pyridin-2-ylmethyl)-2,4-dihydro-3H-1,2,4-triazole-3-thione* (**6a**). Yield 74%. m.p. 224–225 °C. ^1^H-NMR (DMSO-*d*_6_) δ (ppm): 4.11 (s, 2H, CH_2_); 7.05 (d, 1H, CH_arom._, *J* = 10.40 Hz); 7.17–7.21 (dd, 1H, CH_arom._, *J*_1_ = 6.60 Hz, *J*_2_ = 1.50 Hz); 7.26–7.31 (m, 1H, CH_arom._); 7.46 (t, 1H, CH_arom._, *J* = 11.00 Hz); 7.51 (t, 1H, CH_arom._, *J* = 4.40 Hz); 7.61 (tt, 1H, CH_arom._, *J* = 4.56 Hz); 8.39 (d, 1H, CH_arom._, *J* = 5.10 Hz); 13.85 (s, 1H, NH). ^13^C-NMR (DMSO-*d*_6_) δ (ppm): 34.03, 117.40, 119.90, 122.13, 123.40, 129.63, 130.63, 136.64, 149.01, 150.40, 154.59, 155.71, 159.01, 167.97. ESI MS: *m*/*z* = 319.30 [M] (100%).

*4-(5-Chloro-2-methylphenyl)-5-(pyridin-2-ylmethyl)-2,4-dihydro-3H-1,2,4-triazole-3-thione* (**7a**). Yield 73%. m.p. 225 °C. ^1^H-NMR (DMSO-*d*_6_) δ (ppm): 1.81 (s, 3H, CH_3_); 4.00 (s, 2H, CH_2_); 6.95 (d, 1H, CH_arom._, *J* = 10.40 Hz); 7.18–7.22 (dd, 1H, CH_arom._, *J*_1_ = 6.60 Hz, *J*_2_ = 1.80 Hz); 7.25–7.28 (m, 2H, CH_arom._); 7.40–7.43 (dd, 1H, CH_arom._, *J*_1_ = 8.10 Hz, *J*_2_ = 2.10 Hz); 7.59 (tt, 1H, CH_arom._, *J* = 4.56 Hz); 8.39 (d, 1H, CH_arom._, *J* = 5.10 Hz); 13.86 (s, 1H, NH). ^13^C-NMR (DMSO-*d*_6_) δ (ppm): 16.60, 34.11, 122.24, 123.32, 128.43, 129.79, 130.47, 132.21, 133.52, 135.76, 136.63, 149.03, 150.20, 154.37, 167.24. ESI MS: *m*/*z* = 339.00 [M + Na] (100%).

### 3.4. General Procedure for the Synthesis of 5-(Pyridin-2-ylmethyl)-1,3,4-thiadiazol-2-amine Derivatives

*N*-(Substituted-phenyl)-2-(pyridin-2-ylacetyl)hydrazinecarbothioamide (0.01 mol) was mixed for 4 h with concentrated sulfuric acid (0.5 mL). Then to the solution crushed ice was added. After cooling the solution was neutralized with dilute NaOH. The precipitated compound was filtered and then crystallized from ethanol.

*N-(3,4-Dichlorophenyl)-5-(pyridin-2-ylmethyl)-1,3,4-thiadiazol-2-amine* (**1b**). Yield 71%. m.p. 201–202 °C. ^1^H-NMR (DMSO-*d*_6_) δ (ppm): 4.45 (s, 2H, CH_2_); 7.28–7.32 (m, 1H, CH_arom._); 7.42–7.46 (m, 2H, CH_arom._); 7.55 (d, 2H, C_arom._, *J* = 8.70 Hz); 7.78 (tt, 1H, CH_arom._, *J* = 4.56 Hz); 8.05 (d, 1H, CH_arom._, *J* = 2.40 Hz); 8.54 (d, 1H, CH_arom._, *J* = 6.40 Hz); 10.55 (s, 1H, NH). ^13^C-NMR (DMSO-*d*_6_) δ (ppm): 38.01, 117.38, 118.30, 122.31, 122.70, 123.33, 130.78, 131.22, 137.18, 140.55, 149.26, 156.58, 158.08, 164.19. ESI MS: *m*/*z* = 361.00 [M + Na] (100%).

*N-(3-Chloro-4-methylphenyl)-5-(pyridin-2-ylmethyl)-1,3,4-thiadiazol-2-amine* (**2b**). Yield 78%. m.p. 181–182 °C. ^1^H-NMR (DMSO-*d*_6_) δ (ppm): 2.26 (s, 3H, CH_3_); 4.43 (s, 2H, CH_2_); 7.25–7.33 (m, 3H, CH_arom._); 7.43 (d, 1H, CH_arom._, *J* = 8.00 Hz); 7.79 (tt, 1H, CH_arom._, *J* = 4.56 Hz); 7.59 (d, 1H, CH_arom._, *J* = 2.80 Hz); 8.55 (d, 1H, CH_arom._, *J* = 6.40 Hz); 10.31 (s, 1H, NH). ^13^C-NMR (DMSO-*d*_6_) δ (ppm): 18.77, 38.08, 115.98, 117.18, 122.28, 123.32, 128.0, 131.37, 133.31, 137.16, 139.78, 149.27, 156.7, 157.4, 164.52. ESI MS: *m*/*z* = 339.00 [M + Na] (100%).

*5-(Pyridin-2-ylmethyl)-N-[3-(trifluoromethyl)phenyl]-1,3,4-thiadiazol-2-amine* (**3b**). Yield 69%. m.p. 179–180 °C. ^1^H-NMR (DMSO-*d*_6_) δ (ppm): 4.46 (s, 2H, CH_2_); 7.28–7.33 (m, 2H, CH_arom._); 7.44 (d, 1H, CH_arom._, *J* = 10.40 Hz); 7.55 (t, 1H, CH_arom._, *J* = 10.60 Hz); 7.71–7.75 (dd, 1H, CH_arom._, *J*_1_ = 8.40 Hz, *J*_2_ = 1.50 Hz); 7.81 (tt, 1H, CH_arom._, *J* = 4.56 Hz); 8.15 (s, 1H, CH_arom._,); 8.55 (d, 1H, CH_arom._, *J* = 6.60 Hz); 10.59 (s, 1H, NH). ^13^C-NMR (DMSO-*d*_6_) δ (ppm): 38.05, 113.18 (q, 4.20 Hz), 117.75 (q, 4.20 Hz), 120.77, 122.37, 123.35, 125.97 (q, 271.20 Hz), 129.79 (q, 33.20 Hz), 130.14. 137.19, 141.29, 149.27, 156.63, 157.96, 164.43. ESI MS: *m*/*z* = 359.0 [M + Na] (100%).

*N-(3-Bromophenyl)-5-(pyridin-2-ylmethyl)-1,3,4-thiadiazol-2-amine* (**4b**). Yield 76%. m.p. 156–157 °C. ^1^H-NMR (DMSO-*d*_6_) δ (ppm): 4.45 (s, 2H, CH_2_); 6.99–7.03 (m, 1H, CH_arom._); 7.28–7.45 (m, 4H, CH_arom._); 7.78 (tt, 1H, CH_arom._, *J* = 4.56 Hz); 7.86 (t, 1H, CH_arom._, *J* = 2.60 Hz); 8.54 (d, 1H, CH_arom._, *J* = 6.40 Hz); 10.44 (s, 1H, NH). ^13^C-NMR (DMSO-*d*_6_) δ (ppm): 37.31, 116.10, 119.46, 121.92, 122.86, 124.06, 130.88, 138.63, 142.09, 148.15, 148.75, 155.85, 157.38, 164.47. ESI MS: *m*/*z* = 347.20 [M] (100%).

*N-(3-Chlorophenyl)-5-(pyridin-2-ylmethyl)-1,3,4-thiadiazol-2-amine* (**5b**). Yield 78%. m.p. 149–150 °C. ^1^H-NMR (DMSO-*d*_6_) δ (ppm): 4.44 (s, 2H, CH_2_); 6.96–7.08 (m, 1H, CH_arom._); 7.27–7.40 (m, 4H, CH_arom._); 7.79 (tt, 1H, CH_arom._, *J* = 4.56 Hz); 7.85 (t, 1H, CH_arom._, *J* = 2.60 Hz); 8.54 (d, 1H, CH_arom._, *J* = 6.40 Hz); 10.48 (s, 1H, NH). ^13^C-NMR (DMSO-*d*_6_) δ (ppm): 37.94, 115.69, 116.62, 121.12, 122.41, 123.47, 130.58, 133.43, 137.43, 142.00, 149.08, 156.53, 157.74, 164.43. ESI MS: *m*/*z* = 311.90 [M] (100%).

*N-(3-Chloro-4-fluorophenyl)-5-(pyridin-2-ylmethyl)-1,3,4-thiadiazol-2-amine* (**6b**). Yield 75%. m.p. 196–197 °C. ^1^H-NMR (DMSO-*d*_6_) δ (ppm): 4.44 (s, 2H, CH_2_); 6.28–7.45 (m, 4H, CH_arom._); 7.79 (tt, 1H, CH_arom._, *J* = 4.64 Hz); 7.97–7.99 (dd, 1H, CH_arom._, *J*_1_ = 6.00 Hz, *J*_2_ = 2.70 Hz); 8.54 (d, 1H, CH_arom._, *J* = 2.76 Hz); 10.43 (s, 1H, NH). ^13^C-NMR (DMSO-*d*_6_) δ (ppm): 38.06, 116.97 (d, 22.20 Hz), 117.45 (d, 7.80 Hz) 118.38, 119.48 (d, 18.60 Hz), 122.29, 123.32, 137.16, 137.91 (d, 3.30 Hz), 149.26, 153.62 (242.60 Hz), 156.63, 157.68, 164.49. ESI MS: *m*/*z* = 319.30 [M + H] (100%).

*N-(5-Chloro-2-methylphenyl)-5-(pyridin-2-ylmethyl)-1,3,4-thiadiazol-2-amine* (**7b**). Yield 71%. m.p. 145–146 °C. ^1^H-NMR (DMSO-*d*_6_) δ (ppm): 2.24 (s, 3H, CH_3_); 4.53 (s, 2H, CH_2_); 6.99 (dd, 1H, CH_arom._, *J*_1_ = 8.10 Hz, *J*_2_ = 2.10 Hz); 7.22 (d, 1H, CH_arom._, *J* = 10.80 Hz); 7.51 (t, 1H, CH_arom._, *J* = 8.40 Hz); 7.63 (d, 1H, CH_arom._, *J* = 10.00 Hz); 8.02 (tt, 1H, CH_arom._, *J* = 4.48 Hz); 8.20 (d, 1H, CH_arom._, *J* = 2.80 Hz); 8.65 (d, 1H, CH_arom._, *J* = 6.40 Hz); 10.31 (s, 1H, NH). ^13^C-NMR (DMSO-*d*_6_) δ (ppm): 17.62, 36.64, 119.01, 122.21, 123.32, 124.70, 125.99, 130.69, 131.83, 139.48, 140.01, 147.19, 155.23, 157.24, 165.65. ESI MS: *m*/*z* = 334.80 [M] (100%).

### 3.5. 5-(Pyridin-2-ylmethyl)-1,3,4-oxadiazole-2(3H)-thione (c) 

Compound was synthesized and described previously [39,44].

### 3.6. 4-Amino-5-(pyridin-2-ylmethyl)-2,4-dihydro-3H-1,2,4-triazole-3-thione 

5-(Pyridin-2-ylmethyl)-1,3,4-oxadiazole-2(3*H*)-thione (c, 0.01 mole, 1.93 g) was refluxed with 80% hydrazine hydrate (0.5 mL) and ethanol (20 mL) for 4 h. The precipitated compound was filtered and then crystallized from ethanol. Yield 80%. m.p. 210–211 °C. ^1^H-NMR (DMSO-*d*_6_) δ (ppm): 4.20 (s, 2H, CH_2_); 5.5 (br. s, 2H, NH_2_); 7.27 (d, 1H, CH_arom._ *J* = 4.10 Hz); 7.35 (d, 1H, CH_arom._ *J =* 7.80 Hz); 7.75 (tt, 1H, CH_arom._ *J* = 3.43 Hz); 8.45 (d, 1H, CH_arom._ *J* = 3.90 Hz); 13.53 (s, 1H, NH). ^13^C-NMR (DMSO-*d*_6_) δ (ppm): 33.47, 122.32, 123.65, 136.88, 139.40, 149.10, 167, 31, 181.70. ESI MS: *m*/*z* = 206.50 [M − H] (100%).

### 3.7. General Procedure for the Synthesis of 1-(Subtituted phenyl)-3-[3-(pyridin-2-ylmethyl)-5-thioxo-1,5-dihydro-4H-1,2,4-triazol-4-yl]thiourea Derivatives

4-Amino-5-(pyridin-2-ylmethyl)-2,4-dihydro-3*H*-1,2,4-triazole-3-thione (0.01 mol, 2.0 g) and an appropriate isothiocyanate (0.01 mol) were refluxed in acetonitrile for 6 h. The product was filtered and washed carefully with diethyl ether in order to remove unreacted isothiocyanate. The product was then dried and then the residue was purified by column chromatography (eluent: chloroform–methanol 9.5:0.5).

*1-(3,4-Dichlorophenyl)-3-*[3-(pyridin-2-ylmethyl)-5-thioxo-1,5-dihydro-4H-1,2,4-triazol-4-yl]-th*iourea* (**1e**). Yield 68%. m.p. 156–157 °C. ^1^H-NMR (DMSO-*d*_6_) δ (ppm): 4.14 (s, 2H, CH_2_); 7.28 (d, 1H, CH_arom._, *J* = 7.80 Hz); 7.37 (d, 1H, CH_arom._, *J* = 8.50 Hz); 7.48 (m, 1H, CH_arom._); 7.59 (d, 1H, CH_arom._, *J* = 9.00 Hz); 7.56 (tt, 1H, CH_arom._, *J* = 4.08 Hz), 7.68 (m, 1H, CH_arom._); 8.49 (d, 1H, CH_arom._, *J* = 2.80 Hz); 10.78 (br. s, 2H, NH); 13.72 (s, 1H, NH). ^13^C-NMR (DMSO-*d*_6_) δ (ppm): 33.47, 122.35, 122.78, 123.63, 127.25, 130.30, 131.20, 136.71, 136.90, 138.62, 149.11, 151.15, 154.48, 167.25, 181.72. ESI MS: *m*/*z* = 409.90 [M − H] (100%).

*1-(3-chloro-4-methylphenyl)-3-[3-(pyridin-2-ylmethyl)-5-thioxo-1,5-dihydro-4H-1,2,4-triazol-4-yl]thiourea* (**2e**). Yield 65%. m.p. 177–178 °C. ^1^H-NMR (DMSO-*d*_6_) δ (ppm): 2.30 (s, 3H, CH_3_); 4.14 (s, 2H, CH_2_); 7.29 (d, 1H, CH_arom._, *J* = 10.40 Hz); 7.31 (s, 1H, CH_arom._); 7.34 (d, 1H, CH_arom._, *J* = 5.40 Hz); 7.37 (d, 1H, CH_arom._, *J* = 2.40 Hz); 7.40 (d, 1H, CH_arom._, *J* = 10.80 Hz); 7.76 (tt, 1H, CH_arom._, *J* = 4.56 Hz); 8.49 (d, 1H, CH_arom._, *J* = 2.40 Hz); 10.00 (br. s, 1H, NH); 10.59 (br. s, 1H, NH); 13.70 (s, 1H, NH). ^13^C-NMR (DMSO-*d*_6_) δ (ppm): 19.07, 33.42, 116.73, 122.34, 123.62, 127.42, 131.33, 131.41, 133.31, 136.92, 137.52, 149.06, 154.50, 167.26, 181.20. ESI MS: *m*/*z* = 389.00 [M − H] (100%).

*1-[3-(pyridin-2-ylmethyl)-5-thioxo-1,5-dihydro-4H-1,2,4-triazol-4-yl]-3-[3-(trifluoromethyl)-phenyl]thiourea* (**3e**). Yield 80%. m.p. 172–173 °C. ^1^H-NMR (DMSO-*d*_6_) δ (ppm): 4.15 (s, 2H, CH_2_); 2.26 (d, 1H, CH_arom._, *J* = 8.50 Hz); 7.38 (d, 1H, CH_arom._, *J* = 7.80 Hz); 7.49–7.64 (m, 4H, CH_arom._); 7.75 (tt, 1H, CH_arom._, *J* = 9.60 Hz); 8.46 (d, 1H, CH_arom._, *J* = 2.40 Hz); 10.70 (br. s, 2H, NH); 13.83 (s, 1H, NH). ^13^C-NMR (DMSO-*d*_6_) δ (ppm): 33.44, 121.81 (q), 122.32, 123.65 (q), 125.77 (q, 270.20 Hz), 129.66 (q, *J* = 33.2 Hz), 136.88, 139.40, 149.09, 151.07, 154.47, 167.31, 180.31. ESI MS: *m*/*z* = 409.0 [M − H] (100%).

*1-(3-Bromophenyl)-3-[3-(pyridin-2-ylmethyl)-5-thioxo-1,5-dihydro-4H-1,2,4-triazol-4-yl]-thiourea* (**4e**). Yield 75%. m.p. 178–179 °C. ^1^H-NMR (DMSO-*d*_6_) δ (ppm): 4.14 (d, 2H, CH_2_, *J* = 8.40 Hz); 7.29 (m, 6H, CH_arom._); 7.76 (tt, 1H, CH_arom._, *J* = 2.40 Hz); 8.49 (d, 1H, CH_arom._ *J* = 8.40 Hz_._); 10.67 (br. s, 2H, NH); 13.71(s, 1H, NH). ^13^C-NMR (DMSO-*d*_6_) δ (ppm): 34.47, 121.24, 122.37, 123.64, 128.05, 130.43, 136.29, 140.11, 149.12, 151.22, 154.52, 155.2, 167.28, 181.41. ESI MS: *m*/*z* = 429.90 [M − H] (100%)

*1-(3-Chlorophenyl)-3-[3-(pyridin-2-ylmethyl)-5-thioxo-1,5-dihydro-4H-1,2,4-triazol-4-yl]-thiourea* (**5e**). Yield 66%. m.p. 170 °C. ^1^H-NMR (DMSO-*d*_6_) δ (ppm): 4.15 (d, 2H, CH_2_, *J* = 9.00 Hz); 7.14–7.39 (m, 6H, CH_arom._); (tt, 1H, CH_arom._, *J* = 4.56 Hz); 8.47 (d, 1H, CH_arom._, *J* = 5.20 Hz); 10.67 (br. s, 2H, NH); 13.71(s, 1H, NH). ^13^C-NMR (DMSO-*d*_6_) δ (ppm): 34.45, 122.37, 123.81, 125.15, 127.19, 130.15, 132.60, 133.39, 136.87, 137.88, 139.96, 148.84, 151.13, 154.52, 167.98, 181.36. ESI MS: *m*/*z* = 375.00 [M − H] (100%).

*1-(3-Chloro-4-fluorophenyl)-3-[3-(pyridin-2-ylmethyl)-5-thioxo-1,5-dihydro-4H-1,2,4-triazol-4-yl]thiourea* (**6e**). Yield 65%. m.p. 190–192 °C. ^1^H-NMR (DMSO-*d*_6_) δ (ppm): 4.15 (d, 2H, CH_2_, *J* = 9.00 Hz); 7.29 (t, 1H, CH_arom._, *J* = 6.00 Hz); 7.39–7.42 (m, 4H, CH_arom._); 7.75 (tt, 1H, CH_arom._, *J* = 9.00 Hz); 8.49 (d, 1H, CH_arom._, *J* = 6.00 Hz); 10.71 (br. s, 2H, NH); 13.72 (s, 1H, NH). ^13^C-NMR (DMSO-*d*_6_) δ (ppm): 33.43, 116.44 (d, 22.40 Hz), 118.73 (d, 19.40 Hz), 121.22 (d), 121.42, 123.63, 125.08 (d), 135.59 (d), 136.9, 149.09, 152.16, 154.47 (d, 24.30 Hz), 156.37, 167.97, 181.79. ESI MS: *m*/*z* = 393.00 [M − H] (100%).

1*-(2-Chloro-5-methylphenyl)-3-[3-(pyridin-2-ylmethyl)-5-thioxo-1,5-dihydro-4H-1,2,4-triazol-4-yl]thiourea* (**7e**). Yield 63%. m.p. 180–181 °C. ^1^H-NMR (DMSO-*d*_6_) δ (ppm): 2.07 (s, 3H, CH_3_); 4.37 (s, 2H, CH_2_); 7.27 (s, 1H, CH_arom._); 7.65–7.72 (m, 3H, CH_arom._); 8.17 (t, 1H, CH_arom._, *J* = 4.56 Hz); 8.70 (d, 1H, CH_arom._, *J* = 2.40 Hz); 10.37 (br. s, 1H, NH); 10.69 (br. s, 1H, NH); 13.82 (s, 1H, NH). ^13^C-NMR (DMSO-*d*_6_) δ (ppm): 17.41, 29.65, 118.09, 125.25, 126.95, 127.23, 130.47, 132.85, 142.94, 144.76, 148.62, 150.38, 150.87, 167.44, 181.67. ESI MS: *m*/*z* = 389.00 [M − H] (100%).

### 3.8. General Procedure for the Synthesis of N-(Substituted-phenyl)-5-(pyridin-2-ylmethyl)-1,3,4-oxadiazol-2-amine Derivatives 

Triethylamine (2–3 drops) was added to a suspension of thiosemicarbazide (1 mmol) and mercuric chloride (1.25 mmol) in dry DMF (5 mL). The resulting mixture was stirred for a maximum of 8 h at room temperature or until TLC showed the end of the reaction. The suspension was filtered through paper filter, then washed with CHCl_3_. The filtrate was diluted with water, extracted three times with CHCl_3_ (15 mL), the combined organic fractions were dried over MgSO_4_, filtered and concentrated under reduced pressure. The resulting residue was purified by silica gel chromatography (chloroform–methanol; 9.5:0.5).

*N-(3,4-Dichlorophenyl)-5-(pyridin-2-ylmethyl)-1,3,4-oxadiazol-2-amine* (**1f**). Yield 72%. m.p. 198–199 °C. ^1^H-NMR (DMSO-*d*_6_) δ (ppm): 4.36 (s, 2H, CH_2_); 7.28–7.33 (m, 1H, CH_arom._); 7.41–7.45 (dd, 2H, CH_arom._, *J*_1_ = 11.40 Hz, *J*_2_ = 2.70 Hz); 7.55–7.58 (d, 1H, CH_arom._, *J* = 8.70 Hz); 7.71–7.72 (td, 1H, CH_arom._, *J* = 5.70 Hz); 7.85–7.86 (d, 1H, CH_arom._, *J* = 2.40 Hz); 8.49–8.52 (m, 1H, CH_arom._); 10.81 (s, 1H, NH). ^13^C-NMR (DMSO-*d*_6_) δ (ppm): 33.48, 117.11, 117.93, 122.48, 123.05, 123.39, 130.90, 131.31, 137.12, 138.82, 149.37, 154.75, 158.30, 159.44. ESI MS: *m*/*z* = 319.00 [M − H] (100%).

*N-(3-Chloro-4-methylphenyl)-5-(pyridin-2-ylmethyl)-1,3,4-oxadiazol-2-amine* (**2f**). Yield 75%. m.p. 176–177 °C. ^1^H-NMR (DMSO-*d*_6_) δ (ppm): 1.22 (s, 3H, CH_3_); 4.34 (s, 2H, CH_2_); 7.26–7.35 (m, 2H, CH_arom._); 7.38–7.44 (m, 1H, CH_arom._); 7.67–7.68 (d, 1H, CH_arom._, *J* = 0.90 Hz); 7.71-7.72 (td, 1H, CH_arom._, *J* = 2.90 Hz); 7.95 (s, 1H, CH_arom._); 8.50–8.51 (m, 1H, CH_arom._); 10.52 (s, 1H, NH). ^13^C-NMR (DMSO-*d*_6_) δ (ppm): 18.74, 33.50, 115.65, 116.67, 122.47, 123.37, 128.09, 131.49, 133.35, 137.12, 137.88, 149.36, 154.83, 157.98, 159.92 ESI MS: *m*/*z* = 301.20 [M] (100%).

*5-(Pyridin-2-ylmethyl)-N-[3-(trifluoromethyl)phenyl]-1,3,4-oxadiazol-2-amine* (**3f**). Yield 67%. m.p. 145–146 °C. ^1^H-NMR (DMSO-*d*_6_) δ (ppm): 4.37 (s, 2H, CH_2_); 7.29–7.33 (m, 2H, CH_arom._); 7.42–7.45 (m, 1H, CH_arom._); 7.53–7.58 (t, 1H, CH_arom._, *J* = 4.20 Hz); 7.71–7.75 (m, 1H, CH_arom._); 7.77–7.83 (td, 1H, CH_arom._, *J* = 3.90 Hz); 7.97–7.98 (m, 1H, CH_arom._); 8.49–8.52 (dq, 1H, CH_arom._, *J* = 2.40 Hz); 10.83 (s, 1H, NH). ^13^C-NMR (DMSO-*d*_6_) δ (ppm): 33.51, 112.69, 117.90 (q, 4.00 Hz), 120.54 (q, 4.10 Hz), 123.40 (q, 27.40 Hz), 125.91, 130.03 (q, 33.20 Hz), 130.26 (q, 33.20 Hz), 137.13, 139.52, 149.37, 154.80, 158.25, 159.66. ESI MS: *m*/*z* = 319.10 [M − H] (100%).

*N-(3-Bromophenyl)-5-(pyridin-2-ylmethyl)-1,3,4-oxadiazol-2-amine* (**4f**). Yield 70%. m.p. 164–165 °C. ^1^H-NMR (DMSO-*d*_6_) δ (ppm): 4.36 (s, 2H, CH_2_); 7.13–7.17 (m, 1H, CH_arom._); 7.25–7.33 (m, 2H, CH_arom._); 7.42–7.47 (m, 2H, CH_arom._); 7.77–7.84 (m, 2H, CH_arom._, *J* = 3.90 Hz); 8.49–8.52 (m, 1H, CH_arom._); 10.65 (s, 1H, NH). ^13^C-NMR (DMSO-*d*_6_) δ (ppm): 33.51, 115.78, 119.01, 121.94, 122.47, 123.37, 124.19, 130.97, 137.11, 140.30, 149.36, 154.80, 158.14, 159.59. ESI MS: *m*/*z* = 343.10[M − H] (100%).

*N-(3-Chlorophenyl)-5-(pyridin-2-ylmethyl)-1,3,4-oxadiazol-2-amine* (**5f**). Yield 72%. m.p. 147–148 °C. ^1^H-NMR (DMSO-*d*_6_) δ (ppm): 4.36 (s, 2H, CH_2_); 7.00–7.03 (m, 1H, CH_arom._); 7.28–7.45 (m, 4H, CH_arom._); 7.69–7.70 (t, 1H, CH_arom._, *J* = 2.10 Hz); 7.76–7.82 (m, 1H, CH_arom._); 8.50–8.51 (d, 1H, CH_arom._ *J* = 4.80 Hz); 10.67 (s, 1H, NH). ^13^C-NMR (DMSO-*d*_6_) δ (ppm): 33.51, 115.42, 116.20, 121.29, 122.47, 123.38, 130.68, 133.48, 137.11, 140.18, 149.37, 154.81, 158.14, 159.64. ESI MS: *m*/*z* = 329.10 [M − H] (100%).

*N-(3-Chloro-4-fluorophenyl)-5-(pyridin-2-ylmethyl)-1,3,4-oxadiazol-2-amine* (**6f**). Yield 72%. m.p. 178–179 °C. ^1^H-NMR (DMSO-*d*_6_) δ (ppm): 4.35 (s, 2H, CH_2_); 7.28–7.45 (m, 4H, CH_arom._); 7.76-7.22 (m, 2H, CH_arom._); 8.49–8.51 (d, 1H, CH_arom._, *J* = 2.40 Hz); 10.66 (s, 1H, NH). ^13^C-NMR (DMSO-*d*_6_) δ (ppm): 33.52, 117.10, 117.90, 122.47, 123.38, 136.03, 136.06, 137.11, 149.37, 150.60, 153.79, 154.80, 158.12, 159.66. ESI MS: *m*/*z* = 317.10 [M − H] (100%).

*N-(2-Chloro-5-methylphenyl)-5-(pyridin-2-ylmethyl)-1,3,4-oxadiazol-2-amine* (**7f**). Yield 69%. m.p. 215–216 °C. ^1^H-NMR (DMSO-*d*_6_) δ (ppm): 3.36 (bs, 3H, CH_3_); 4.36 (s, 2H, CH_2_); 7.28–7.33 (m, 1H, CH_arom._); 7.41–7.45 (m, 2H, CH_arom._); 7.55–7.57 (d, 1H, CH_arom._, *J* = 4.30 Hz); 7.77–7.82 (td, 1H, CH_arom._, *J* = 3.90 Hz); 7.85–7.86 (d, 1H, CH_arom._, *J* = 1.20 Hz); 8.49–8.52 (m, 1H, CH_arom._). ^13^C-NMR (DMSO-*d*_6_) δ (ppm): 33.49, 33.51, 117.14, 117.95, 122.49, 123.03, 123.40, 130.91, 131.31, 137.14, 138.87, 149.37, 154.37, 158.30, 159.48. ESI MS: *m*/*z* = 301.20 [M] (100%).

### 3.9. Biological Assays

#### 3.9.1. In Vitro Evaluation of Antimicrobial Activity

Microorganisms used in this study were as follows: Gram-positive bacteria: *S. aureus* NCTC 4163, *S. aureus* ATCC 25923, *S. aureus* ATCC 6538, *S. aureus* ATCC 29213, *S. epidermidis* ATCC 12228, *S. epidermidis* ATCC 35984, *Enterococcus hirae* ATCC 10541, *Enterococcus faecalis* ATCC 29212, *Bacillus subtilis* ATCC 6633, *Bacillus cereus* ATCC 11778, *Micrococcus luteus* ATCC 9341, *M. luteus* ATCC 10240; Gram-negative rods: *E. coli* ATCC 10538, *E. coli* ATCC 25922, *E. coli* NCTC 8196, *P. vulgaris* NCTC 4635, *P. aeruginosa* ATCC 15442, *P. aeruginosa* NCTC 6749, *P. aeruginosa* ATCC 27863, *B. bronchiseptica* ATCC 4617 and yeasts: *C. albicans* ATCC 10231, *C. albicans* ATCC 90028, *C. parapsilosis* ATCC 22019. All of listed microorganisms used were obtained from the collection of the Department of Pharmaceutical Microbiology, Medical University of Warsaw, Poland.

#### 3.9.2. Media, Growth Conditions and Antimicrobial Activity Assays

Antibacterial activity was examined by the disc-diffusion method under standard conditions using Mueller-Hinton II agar medium (Becton Dickinson, Franklin Lakes, NJ, USA) according to CLSI (previously NCCLS) guidelines [41]. Antifungal activities were assessed using Mueller-Hinton agar 2% glucose and 0.5 mg/ml Methylene Blue Dye Medium [41]. Sterile filter paper discs (9 mm diameter, Whatman No 3 chromatography paper) were dripped with tested compound solutions (in DMSO) to load 400 mg of a given compound per disc. Dry discs were placed on the surface of appropriate agar medium. The results (diameter of the growth inhibition zone) were read after 18 h of incubation at 35 °C.

#### 3.9.3. Cytotoxicity and Antiviral Assays

CD4+ human T-cells containing an integrated HTLV-1 genome (MT-4) were purchased from American Type Culture Collection (ATCC). Laboratory strain IIIB of Human Immunodeficiency Virus type-1 (HIV-1) was obtained from the supernatant of the persistently infected H9/IIIB cells (NIH 1983). Compounds’ activity against HIV-1 was based on inhibition of virus-induced cytopathogenicity in MT-4 cell acutely infected with a multiplicity of infection (MOI) of 0.01, as described in Drzewiecka et al. [45]. After a 4-day incubation at 37 °C, cell viability was determined by the MTT method [46]. Efavirenz was used as reference inhibitor.

## 4. Conclusions

We successfully synthesized five groups of compounds: thiosemicarbazides **1**–**7**, 1,2,4-triazoles **1a**–**7a**, 1,3,4-thiadiazoles **1b**–**7b**, 1,3,4-oxadiazoles **1f**–**7f** and thiourea derivatives **1e**–**7e** to check their microbiological activity, cytotoxicity and anti HIV activity. Introduction of the 1,2,4-triazole, thiadiazole and oxazole ring to a 2-pyridyl moiety by a methylene linker decreased the antimicrobial activity and cytotoxicity. A second substituent like a phenyl ring connected with halogen atoms (chlorine, bromine or fluorine) had no influence on the biological activity. It could be summarized that all obtained derivatives possess moderate activity and their structure-activity relationships need to be evaluated.

## Figures and Tables

**Table 1:** Crystal data and structure refinement parameters for **3**, **3b**, **7a** and **7f**.

Compound	3	3b	7a	7f
Empirical formula	C_15_H_13_F_3_N_4_OS	C_15_H_11_F_3_N_4_S	C_16_H_14_ClN_3_S	C_15_H_13_ClN_4_O
Formula weight	354.35	336.34	315.81	300.74
Temperature	80(2) K	100(2) K	120(2) K	295(2) K
Wavelength (Å)	0.71073	0.71073	1.54184	1.54184
Crystal system	monoclinic	monoclinic	triclinic	monoclinic
Space group	*P*2_1_/*n*	*P*2_1_/*c*	*P*-1	*I*2/*a*
Unit cell dimensions				
a (Å)	4.6532(9)	16.152(5)	7.417(2)	15.738(2)
b (Å)	34.942(18)	7.668(2)	8.601(2)	13.945(2)
c (Å)	9.800(3)	11.594(4)	12.111(3)	13.254(2)
α (°)	90	90	106.72(2)	90
β (°)	91.17(5)	90.99(3)	92.49(2)	90.94(1)
γ (°)	90	90	95.86(2)	90
Volume (Å^−3^)	1593.1(10)	1435.7(8)	733.9(3)	2908.4(7)
Z	4	4	2	8
F(000)	728	688	328	1248
Density (calcd) (g/cm^3^)	1.477	1.556	1.429	1.374
Absorpt. coeff. (mm^-1^)	0.246	0.263	3.591	2.364
Max. and min. transmission	0.985 and 0.8996	0.9844 and 0.8886	0.990 and 0.9833	0.798 and 0.6493
Crystal size (mm)	0.44 × 0.07 × 0.06	0.46 × 0.13 × 0.06	0.22 × 0.20 × 0.18	0.20 × 0.20 × 0.10
θ range for data coll. (°)	2.72 to 30.39°.	2.94 to 28.75°.	3.82 to 73.65°.	4.24 to 73.95°.
Index ranges	−5 ≤ *h* ≤ 5, −49 ≤ *k* ≤ 49, −12 ≤ *l* ≤ 13	−21 ≤ *h* ≤ 21, −9 ≤ *k* ≤ 10, −11 ≤ *l* ≤ 15	−9 ≤ *h* ≤ 9, −10 ≤ *k* ≤ 10, −11 ≤ *l* ≤ 15	−13 ≤ *h* ≤ 19, −16 ≤ *k* ≤ 11, −16 ≤ *l* ≤ 12
Reflections collected	8587	8611	4813	3609
Independent reflections	4085 [R(int) = 0.0466]	3364 [R(int) = 0.0353]	2849 [R(int) = 0.0251]	2419 [R(int) = 0.0189]
Data/parameters	4085/229	3364/239	2849/195	2419/196
Goodness-of-fit on F^2^	1.109	1.061	1.086	1.049
Final R indices [I > 2σ (I)]	R1 = 0.0594 wR2 = 0.1229	R1 = 0.0398 wR2 = 0.0956	R1 = 0.0432 wR2 = 0.1012	R1 = 0.0448 wR2 = 0.1240
R indices (all data)	R1 = 0.0871 wR2 = 0.1354	R1 = 0.0600 wR2 = 0.1001	R1 = 0.0490 wR2 = 0.1050	R1 = 0.0580 wR2 = 0.1382
Extinction coefficient	--	--	--	0.0017(2)
Δρmax/min (e Å^−3^)	0.38/−0.38	0.32/−0.24	0.58/−0.33	0.18/−0.29
CCDC No.	1539080	1539081	1539083	1539082

**Table 2:** Cytotoxicity and anti-HIV-1 activity of compounds (**1**–**7**, **1a**–**7a** and **1b**–**7b**).

Compounds	MT-4	HIV-1_IIIB_
	^a^ CC_50_	^b^ EC_50_
	(μM)	
**1**	>100	>100
**2**	>100	>100
**3**	>100	>100
**4**	>100	>100
**5**	>100	>100
**6**	>100	>100
**7**	>100	>100
**1a**	>100	>100
**2a**	>100	>100
**3a**	>100	>100
**4a**	>100	>100
**5a**	>100	>100
**6a**	>100	>100
**7a**	>100	>100
**1b**	>100	>100
**2b**	47	>47
**3b**	70.6	>70.6
**4b**	74.0	>74.0
**5b**	>100	>100
**6b**	>100	>100
**7b**	>100	>100
EFV	45.0	0.003

^a^ Compound concentration (µM) required to reduce the viability of mock-infected MT-4 cells by 50%, as determined by the MTT method; ^b^ Compound concentration (μM) required to achieve 50% protection of MT-4 cells from the HIV-1 induced cytopathogenicity, as determined by the MTT method.

## Supplementary Materials

Supplementary Materials are available online.
