# Supplementary material for: Synthesis and Biological Activities of Ethyl 2-(2-pyridylacetate) Derivatives Containing Thiourea, 1,2,4-triazole, Thiadiazole and Oxadiazole Moieties
Source: Molecules. 2017 Mar 6;22(3):409. doi: 10.3390/molecules22030409 (PMC6155191; doi:10.3390/molecules22030409)
Supplement: Supplementary file 1 [file molecules-22-00409-s001.pdf]

Supplementary Materials: Article Synthesis and Biological Activities of Ethyl 2-(2-pyridylacetate) Derivatives Containing Thiourea, 1,2,4-triazole, Thiadiazole and Oxadiazole Moieties

Daniel Szulczyk [1,2,*], Piotr Tomaszewski [3], Michał Jóźwiak [1,2,3], Anna E. Kozioł [4], Tadeusz Lis [5], David Collu [6], Filippo Iuliano [7] and Marta Struga [1,2]

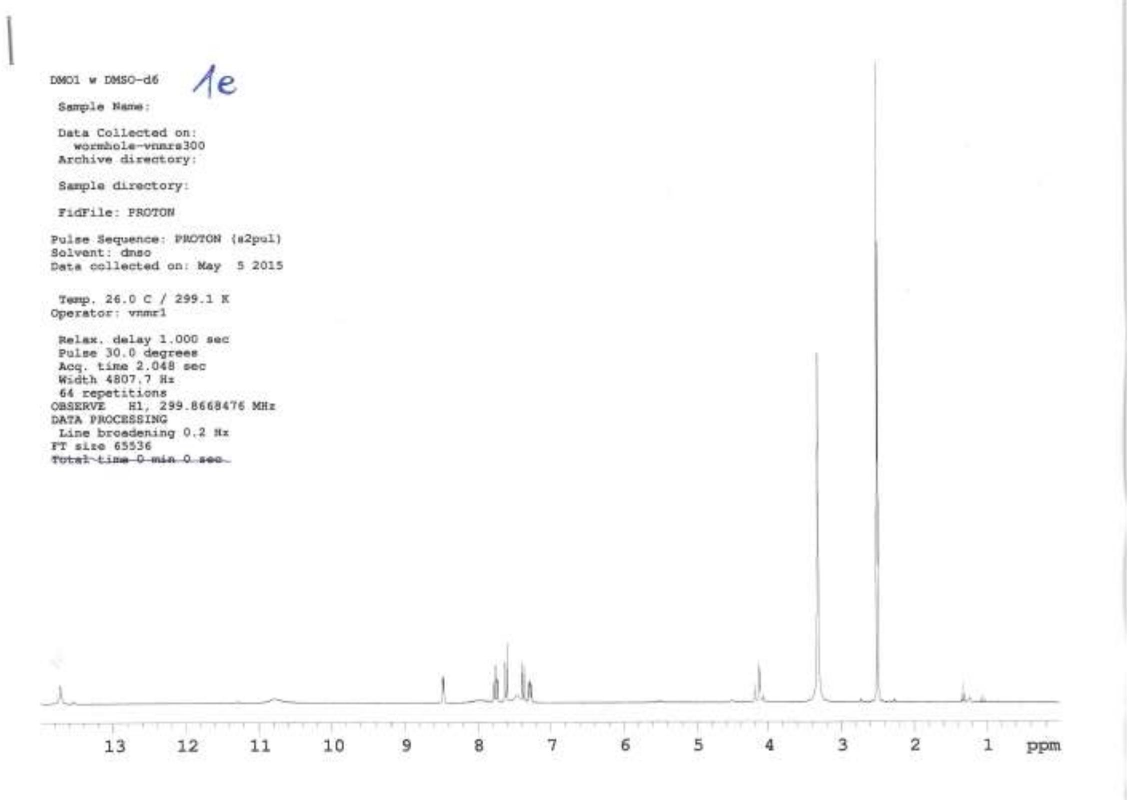

Figure S1. ^{1}H-NMR-1e-Sample.

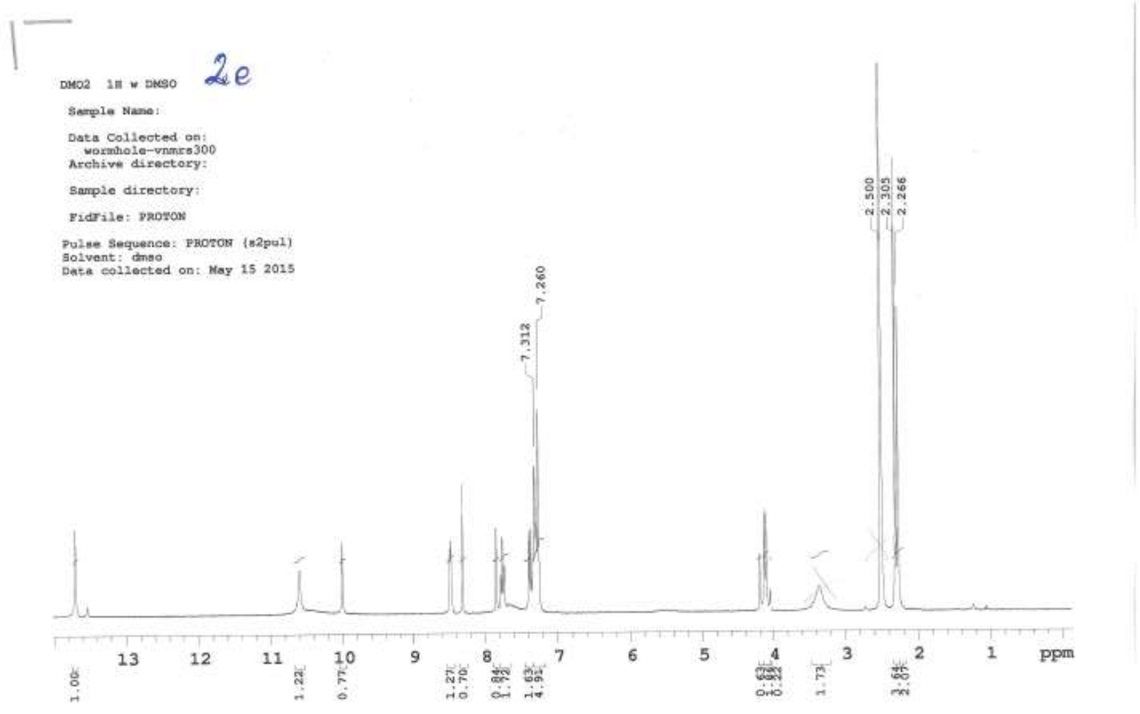

Figure S2. ^{1}H-NMR-2e-Sample.

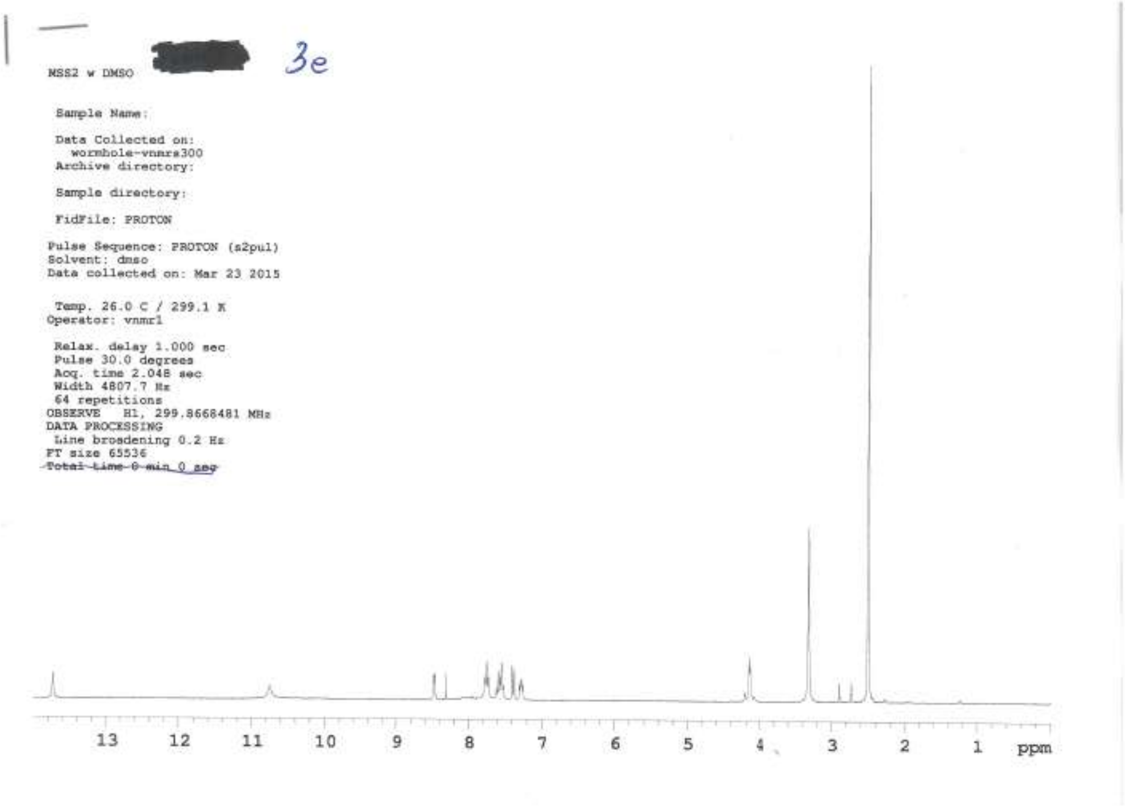

Figure S3. ^{1}H-NMR-3e-Sample.

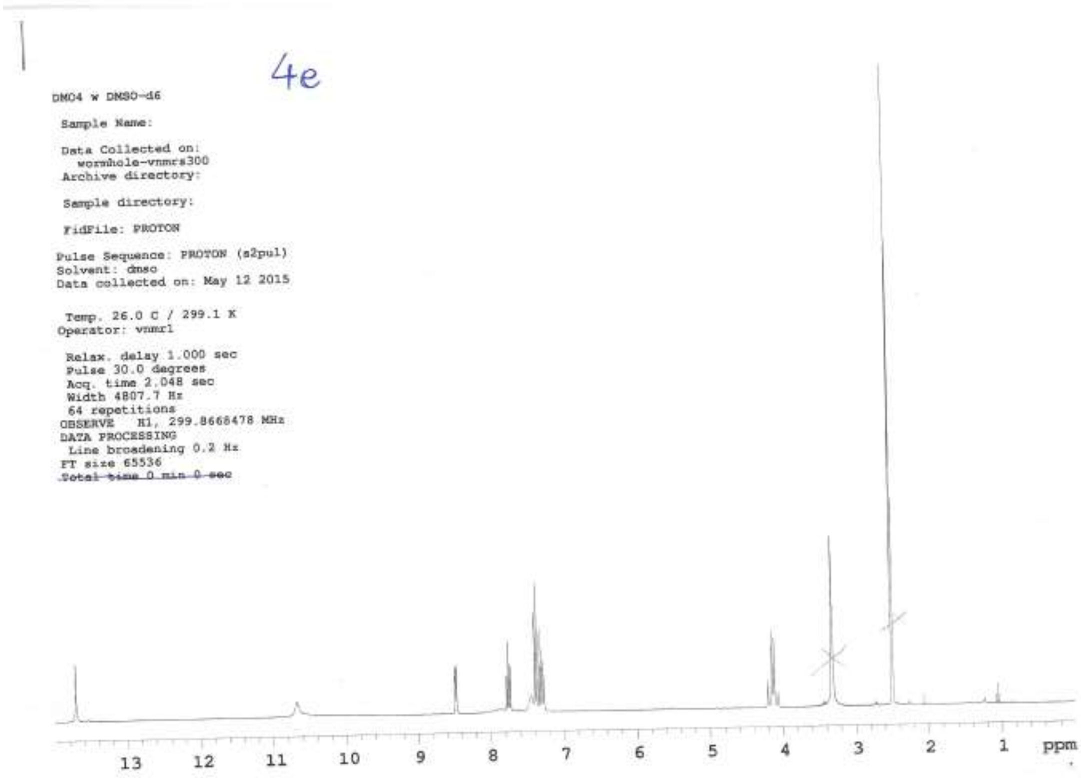

Figure S4. ^{1}H-NMR-4e-Sample.

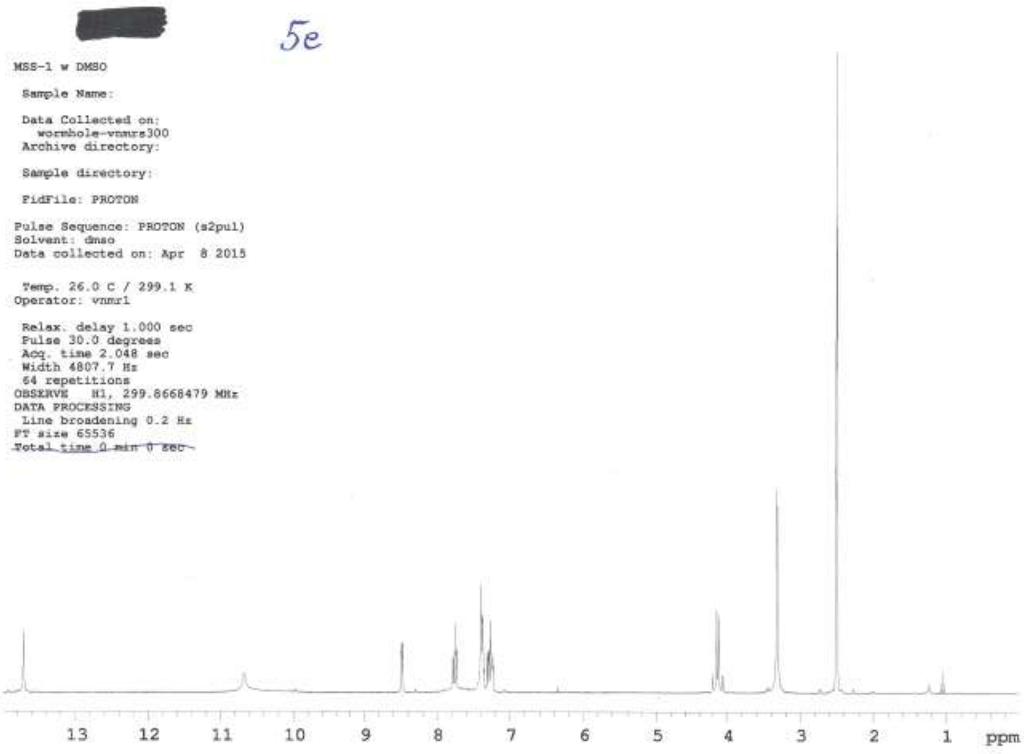

Figure S5. ^{1}H-NMR-5e-Sample.

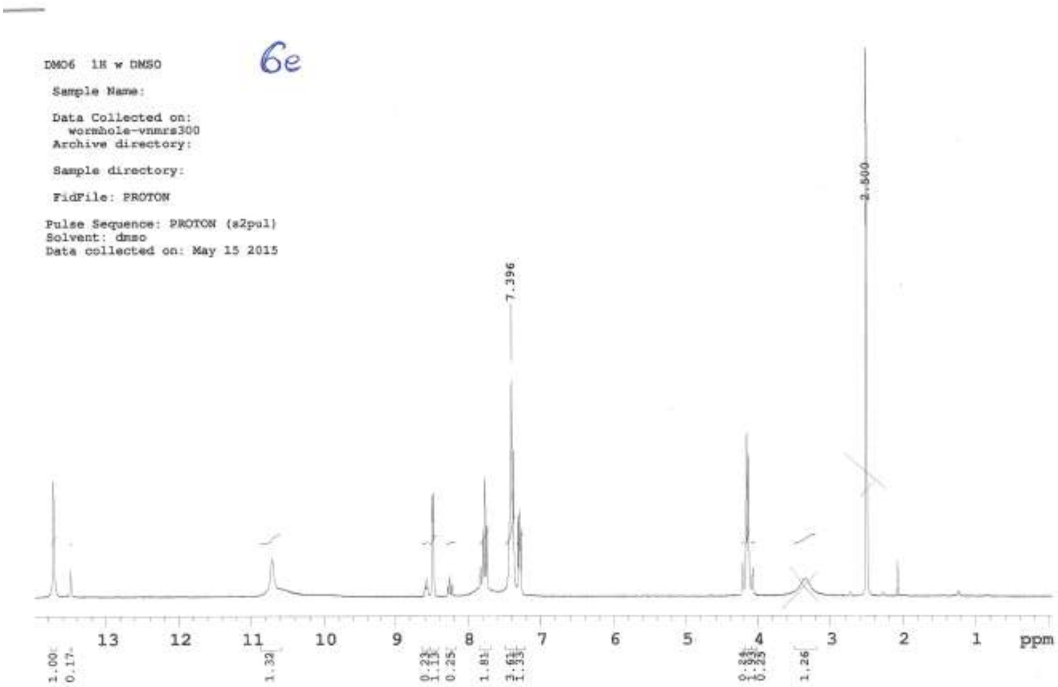

Figure S6. ^{1}H-NMR-6e-Sample.

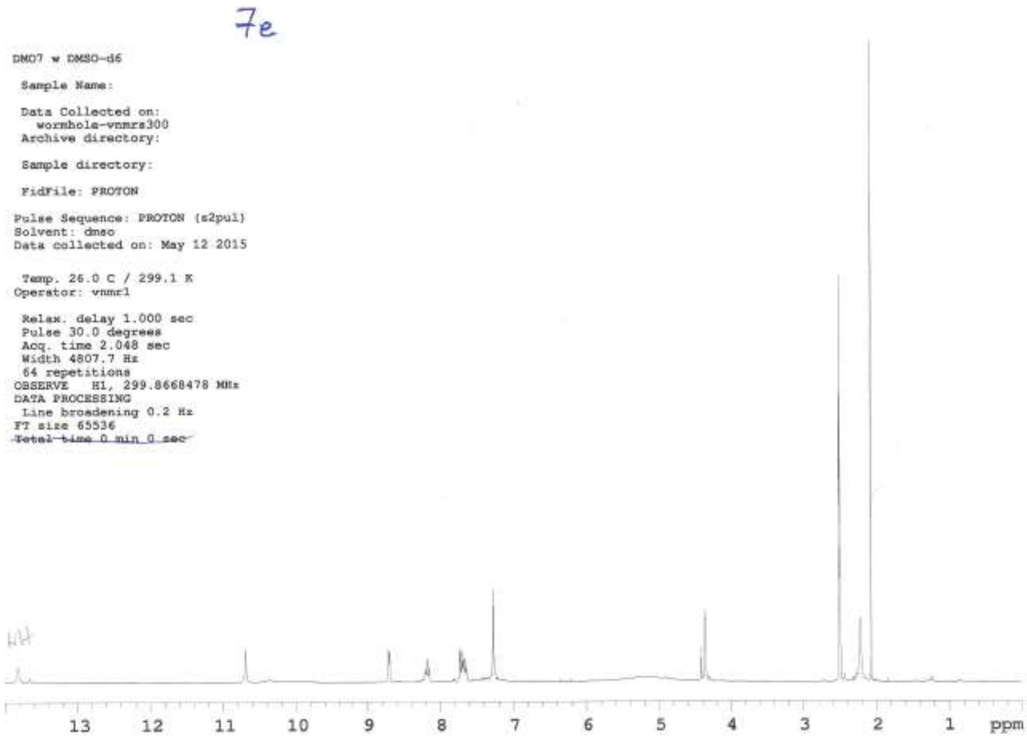

Figure S7. ^{1}H-NMR-7e-Sample.

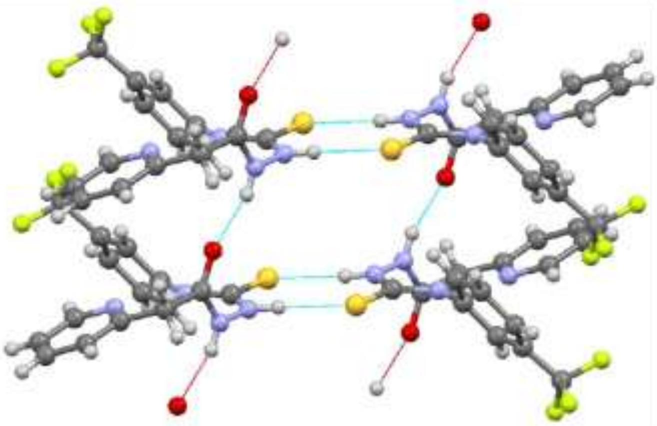	2-(pyridin-2-ylacetyl)-N-[3-(trifluoromethyl)phenyl]hydrazinecarbothioamide (**3**)<br>Hydrogen bonds:<br>NH..S (thiourea...thiourea) stabilize dimers<br>NH...O (amide...amide) form chains<br><br>Resulting association:<br>tape with hydrophobic groups oriented outside
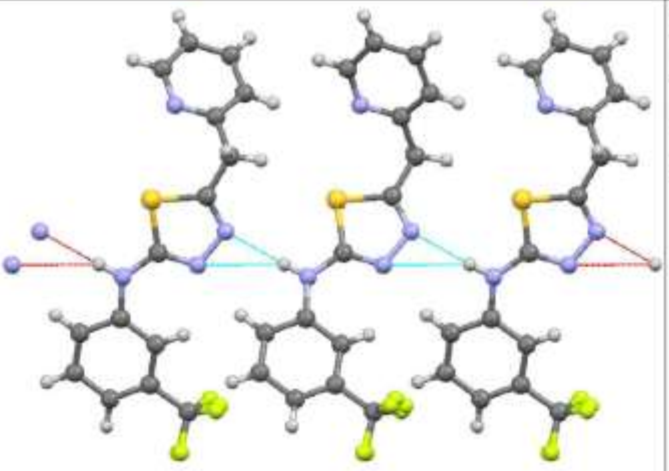	5-(pyridin-2-ylmethyl)-N-[3-(trifluoromethyl)phenyl]-1,3,4-thiadiazol-2-amine (**3b**)<br><br>NH...N hydrogen bonds join molecules into chains
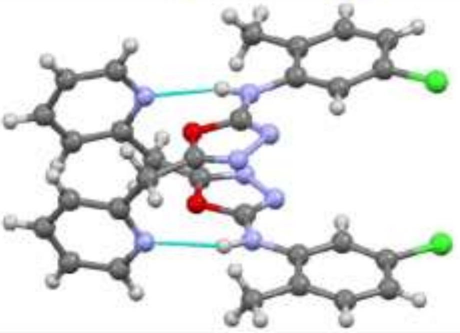	N-(2-chloro-5-methylphenyl)-5-(pyridin-2-ylmethyl)-1,3,4-oxadiazol-2-amine (**7f**)<br><br>NH...N hydrogen bonds (amine...pyridyl) form dimer with crystallographic symmetry of two-fold axis
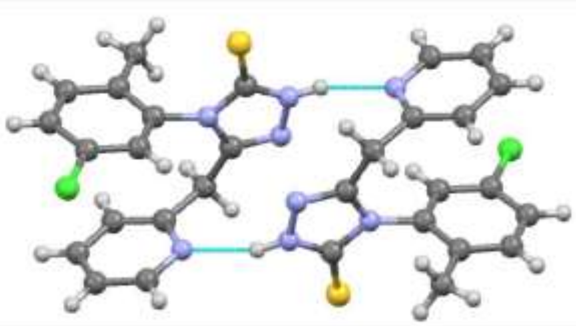	4-(5-chloro-2-methylphenyl)-5-(pyridin-2-ylmethyl)-2,4-dihydro-3H-1,2,4-triazole-3-thione (**7a**)<br><br>NH...N (triazole...pyridyl) centrosymmetric dimer

Figure S8. Intermolecular hydrogen bonds.
